# The first hypothelminorheic Crustacea (Amphipoda, Dogielinotidae, *Hyalella*) from South America 

**DOI:** 10.3897/zookeys.236.3930

**Published:** 2012-10-02

**Authors:** Stella Gomes Rodrigues, Alessandra Angélica de Pádua Bueno, Rodrigo Lopes Ferreira

**Affiliations:** 1 Universidade Federal de Lavras, Departamento de Biologia, Setor de Zoologia, Programa de Pós-Graduação em Ecologia Aplicada, Campus Universitário, 37200-000, Lavras, Minas Gerais, Brazil

**Keywords:** Amphipoda, hypothelminorheic, *Hyalella*, biodiversity, conservation

## Abstract

Most of known troglobiotic species occur in caves and subterranean environments from great depths. However, recently more attention has been given to other subterranean environments, such as the hypothelminorheic habitats. It comprises the most superficial among all subterranean habitats. This kind of environment is characterized by the constant presence of wet spots, absence of light and very particular abiotic characteristics, comprising unique species. The first hypothelminorheic Amphipoda from South America is here described, a new species of the genus *Hyalella* which occurs in a wetland on Southern Brazil. The new species differs from other troglobiotics of the genus by the presence of a curved seta on the inner ramus of uropod 1 and elongation of appendices, as the first pair of antennae and peraeopods 6 and 7. However, human impacts in the area where the new species occurs have changed heavily their habitat, which may have led the species to a critical level of threat or even extinction, demonstrating the fragility of this environment.

## Introduction

For decades cave organisms, especially those more adapted, were thought to be associated mainly to deep portions of caves. Although it was known that the subterranean environment was much wider than macro-caves, the study of cave fauna has been historically focused on them. There are many studies concerning other subterranean habitats, especially the MSS (“*Milleu Souterrain Superficiel*”), and many troglobiotic species (exclusively subterranean-dwellers) were found in such habitats (Juberthie et al. 1980, Racovitza 1983, Juberthie and Decu 1994, Růžička 1999, Juberthie 2000, Culver and Pipan 2009a, 2009b). However, recently more attention has been given to superficial subterranean compartments, which are generally called “shallow subterranean habitats” (Culver and Pipan 2009a).

Among a great variety of such habitats, there is the hypothelminorheic habitat. This habitat comprises the most superficial subterranean habitat. It is characterized by the presence of persistent wet spots fed by subterranean water in depressions of moderately sloped areas (Culver et al. 2006, Culver and Pipan 2009a). There is no light in such habitats, like in caves; physical and chemical properties are unique, such as the abundance of organic matter, drainage area of less than 10.000 m^2^, high conductivity and lower annual variation of temperature when compared with surface waters (Culver et al. 2006). Hypothelminorheic habitats may comprise species unique to this habitat or common to other subterranean environment (Culver and Pipan 2009a).

Amphipods are among the species that may be found in hypothelminorheic habitats (Fiser et al. 2010). In Europe and North America, there are species known to be restricted to such habitats. However, there are no records of hypothelminorheic species from South America, especially due to the lack of studies concerning this kind of habitat. The Neotropics comprises about 7% of described freshwater species, and the diversity of South America in family, genus and species levels is relatively low when compared with other regions of the planet (Väinölä et al. 2008).

Freshwater South American amphipods belong to the families Dogielinotidae, Bogidiellidae, Ingolfiellidae, Phreatogammaridae, Pontogeneiidae and Paraleptamphopidae. However, in South America, these families are restricted to subterranean environments, except Dogielinotidae, and most of them comprise few species. On the other hand, Dogielinotidae is widespread, the genus *Hyalella* Smith, 1874 is found in many different epigean habitats, besides some troglobitic species that occur in caves (Grosso and Peralta 1999).

At present, there are 56 described species for this genus, of which 14 occur in Brazil (Bastos-Pereira and Bueno 2012). Two of them are troglobiotic and both occur in caves of the São Paulo state, Southeastern of the country (Cardoso et al. 2011). Certainly the diversity of *Hyalella* is underrepresented, particularly with respect to species that occur in subterranean environments, what results from few studies concerning the genus (Väinölä et al. 2008).

The aim of this work is to describe the first hypothelminorheic *Hyalella*, which also represents the first hypothelminorheic species described for South America. We also discuss about the habitat loss of this new species, considering even the possibility of its extinction due to anthropogenic actions.

## Material and methods

The specimens were collected in July 2002, on a wetland in the municipality of Roque Gonzales, Rio Grande do Sul state, Southern Brazil. The sampling was made with the aid of a handnet, which collected the sediment, water column and the riparian vegetation.

Body and head length of the animals were measured through an optic microscope with a milimetric scale. The body measurement was made from the tip of the head to the base of telson. Ten animals (five males and five females) were dissected and the appendices were mounted on permanent slides, which were used to illustrate the new species.

The description was made based on main morphological characteristics of Brazilian species of *Hyalella*, such as the gnathopods, uropods and telson, according to González et al. (2003a, 2003b), González et al. (2006), Cardoso et al. (2011), and Bastos-Pereira and Bueno (2012). The terminology used for setae of the appendices follows Zimmer et al. (2009).

Type material is deposited on Coleção de Crustáceos da Universidade Federal de Lavras (UFLA) and Museu Nacional do Rio de Janeiro (MNRJ).

The pictures of fixed animals were taken through a stereomicroscope Zeiss Stemi 2000-C connected to a photographic camera, using Carl Zeiss AxioVision program SE64 Rel 4.8.3. The images from the site of occurrence of new species were taken from Google Earth.

## Systematics

### Order Amphipoda Latreille, 1816

### Suborder Gammaridea Latreille, 1802

### Family Dogielinotidae Gurjanova, 1953

### Genus *Hyalella* S. I. Smith, 1874

#### Hyalella imbya

Rodrigues & Bueno sp. n.

urn:lsid:zoobank.org:act:F065930A-43A1-453F-8785-CCBD576E6C73

http://species-id.net/wiki/Hyalella_imbya

**Figure 1.**
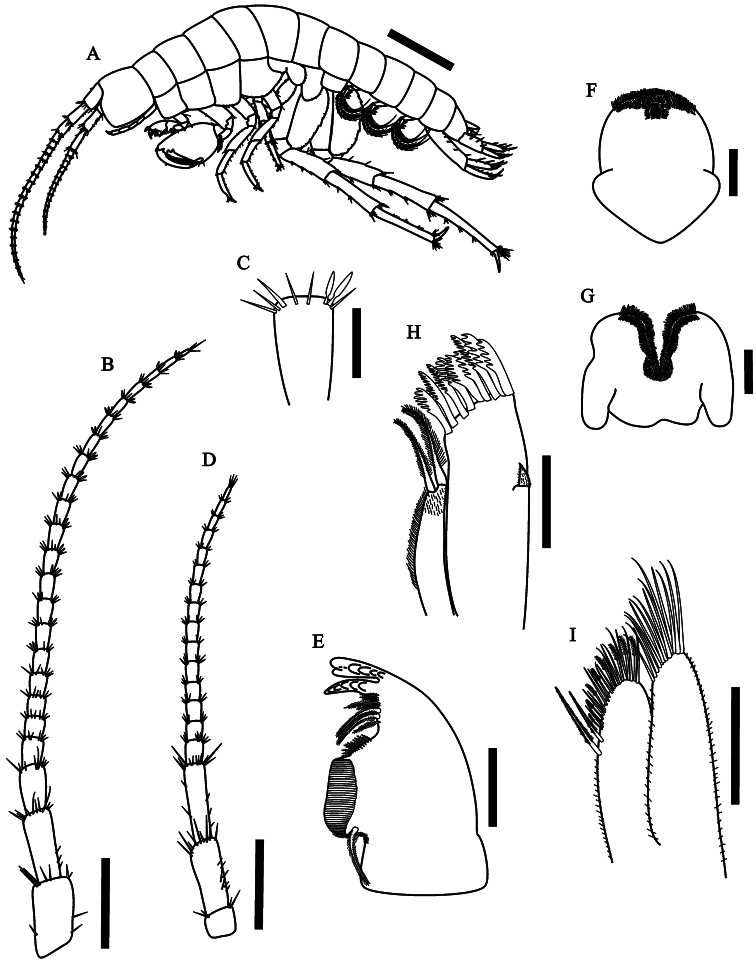
*Hyalella imbya* sp. n. Rodrigues and Bueno (male paratype, UFLA 0187). **A** habitus from holotype **B** antenna 1 **C** antenna 1 article showing two aesthetascs **D** antenna 2 **E** left mandible **F** upper lip **G** lower lip **H** maxilla 1 **I** maxilla 2. Scale bar equal 1 mm for **A**; 500 µm for **B**; 100 µm for **C–I**.

Figs 1 [Fig F2] [Fig F3] [Fig F4] –5

##### Type material. 

Holotype: male, Brazil, Rio Grande do Sul state, Roque Gonzales municipality, wetland, Ijuí watershed, Uruguay hydrographic region, (28°13'55.6"S, 54°58'37.3"W) (MNRJ 23384), allotype female (MNRJ 23385), July, 7, 2002, Stenert, C. coll.

##### Paratypes.

MNRJ 23386 (5 males; 5 females; 5 juveniles), UFLA 0187 (10 males; 10 females; 10 juveniles) with the same data as the holotype.

##### Type locality.

Brazil, Rio Grande do Sul state: Roque Gonzales municipality, 28°13'55.6"S, 54°58'37.3"W, wetland, Ijuí watershed, Uruguay hydrographic region, ca 200 m high, July 7 2002, Stenert, C. coll.

##### Diagnosis.

Body surface smooth. Eyes absent. Antenna 1 longer than antenna 2, flagellum with 18–23 articles. Antenna 2 less than half body length, flagellum with 14-16 articles. Maxilliped with distal nail longer than dactylus. Gnathopod 1 propodus length less than twice maximum width, hammer shape, inner face with 7 pappose setae, without comb-scales. Gnathopod 2 carpus wider than long, posterior lobe elongated without comb-scales or denticles in the border; propodus ovate, without comb-scales, palm sub-equal to posterior margin, slope oblique, palm with two rows of several cuspidate setae with an accessory setae and simple setae. Peraeopod 5 smaller than others; peraeopod 6 and 7 much more longer than others. Uropod 1 inner ramus of male with a short curved seta, four cuspidate setae with an accessory seta apically, with one of them almost half length of the outer ramus. Uropod 3 shorter than telson, peduncle wider than ramus, with one cuspidate seta with an accessory seta distally. Telson wider than long, with two long simple apical setae. Sternal gills present on segments 3 to 7.

##### Description of male.

Mean body length: 5.03 ± 0.85 mm, mean head length: 0.46 ± 0.07 mm (n=10). Body surface smooth; epimeral plates not acuminate (Fig. 1A, Fig. 6A).

Head smaller than 2 thoracic segments, rostrum absent. Eyes absent (Fig. 6B).

Antenna 1 (Fig. 1B) longer than antenna 2, more than half body length; peduncle surpassing head length; flagellum with 18 to 23 articles; aesthetascs (Fig. 1C) ocurring in pairs distally on flagellum after article 5.

Antenna 2 (Fig. 1D) peduncle not surpassing the second pereionite, less than half body length, peduncle slender, longer than head; flagellum with 14 to 16 articles, longer than peduncle.

Mandible basic amphipodan (in the sense of Watling 1993), but without palp; incisor toothed; left lacinia mobilis with six teeth (Fig. 1E); seta row on left mandible with five main pappose setae plus accessory setae, right mandible with three main pappose setae plus accesory setae; molar large, cylindrical and triturative with setules around its circumference.

Upper lip (Fig. 1F) margin rounded; distal border covered by setules on dorsal and ventral faces.

Lower lip (Fig. 1G) outer lobes rounded, with setules on dorsal and ventral faces.

Maxilla 1 (Fig. 1H) palp uniarticulate, short, longer than wide, covered by several simple setae and reaching less than half length the distance between the base of palp and tip of setae on outer plate. Inner plate slender, shorter than outer plate, with two long papposerrate apical setae, several simple setae on inner margin; outer plate with 8–9 long serrate setae.

Maxilla 2 (Fig. 1I) inner plate shorter than outer plate, with two long papposerrate, eight serrulate and several simple apical setae; outer plate with abundant long simple setae; outer and inner plates with several setules.

Maxilliped (Fig. 4B) inner plate with three strong cuspidate setae apically, several pappose setae on apical and inner borders, inner plate recovered by abundant short setule; outer plate larger than inner plate, recovered by setule and with three pappose setae and several simple setae; palp longer than outer plate, four articles; article 1 wider than long, outer and inner faces with short simple setae; article 2 wider than long, inner face with several long simple setae; article 3 wider than long, outer and inner faces with several long simple setae and outer face with four pappose setae; dactylus unguiform recovered by short simple setae, shorter than third article, inner border with several simple setae; distal nail longer than dactylus.

**Figure 2. F2:**
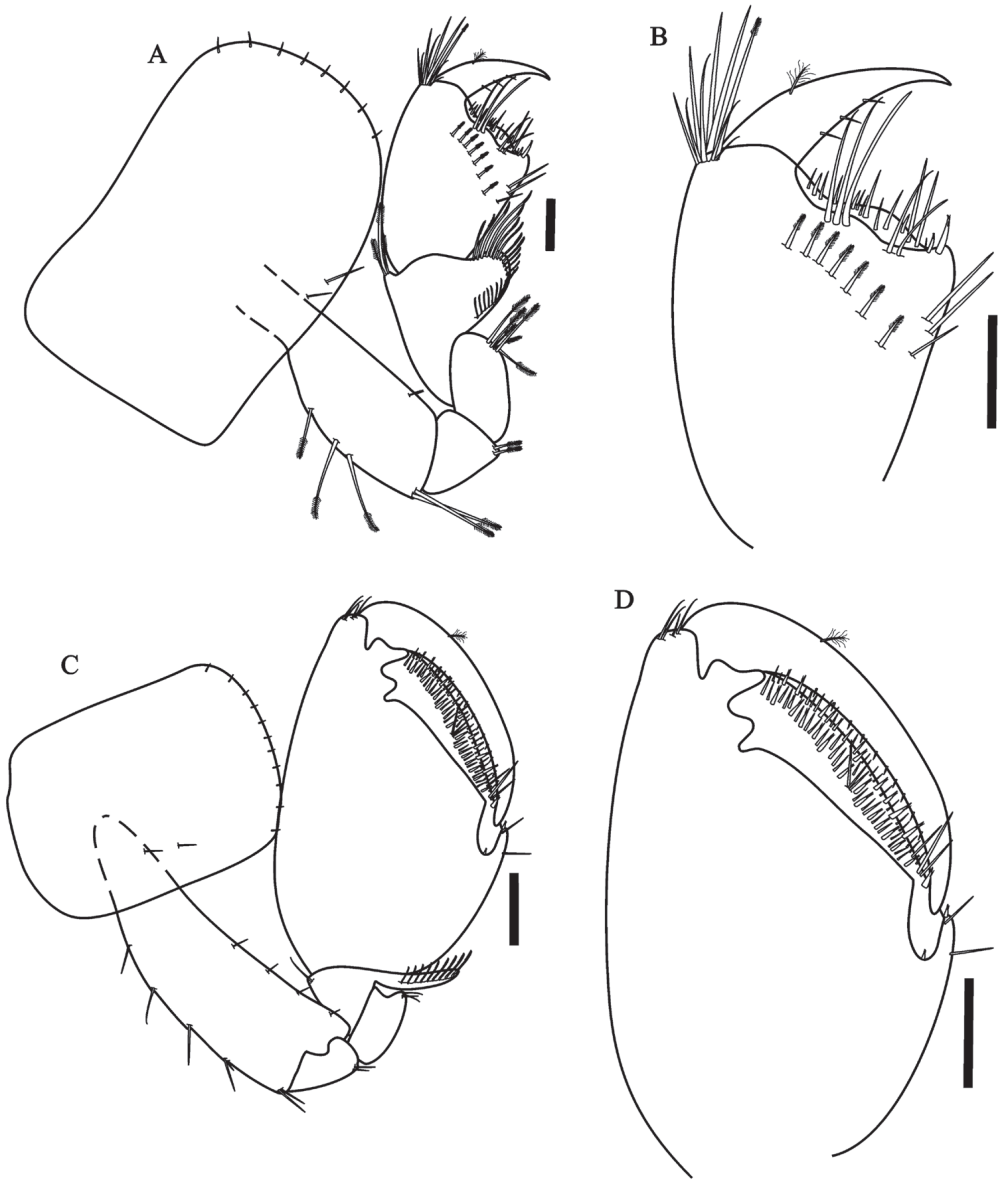
*Hyalella imbya* sp. n. Rodrigues and Bueno (male paratype, UFLA 0187). **A** gnathopod 1 **B** gnathopod 1 propodus and dactylus **C** gnathopod 2 **D** gnathopod 2 propodus and dactylus. Scale bars equals 100 µm for **A–D**.

Gnathopod 1 (Fig. 2A) subchelate; coxal plate wider than long, with simple setae on the border; basis, ischium and merus with serrate setae dorsally; carpus longer than wide, shorter than propodus, with lateral distal lobe produced and forming a scoop-like structure, border pectinate with several serrate setae, without denticles and comb-scales in their basis; border propodus width 3/4 of maximum length, hammer-shaped (Fig. 2B), without setae on anterior border, without comb-scales, inner face with 7 serrate setae, with simple setae on the disto-posterior border; palm slope transverse, margin slightly concave, posterior distal corner with two cuspidate setae with an accessory seta; dactylus claw-like without comb-scales, one plumose seta dorsally and few setae ventrally.

Gnathopod 2 (Fig. 2C) subchelate; basis hind margin with five groups of simple setae; merus with few setae on posterior margin; carpus wider than long, posterior lobe slim produced between merus and propodus, border pectinate with several short serrate setae, without denticles or comb-scales; propodus ovate (Fig. 2D), length 1.4 maximum width, without comb-scales; palm sub-equal than posterior margin of propodus, slope oblique, palm with two rows of several cuspidate setae with an accessory setae and simple setae, posterior distal corner with few simple setae and with a cup for dactylus; dactylus claw-like, congruent with palm, with few endal setae and a plumose seta dorsally, few setae ventrally, without comb-scales.

**Figure 3. F3:**
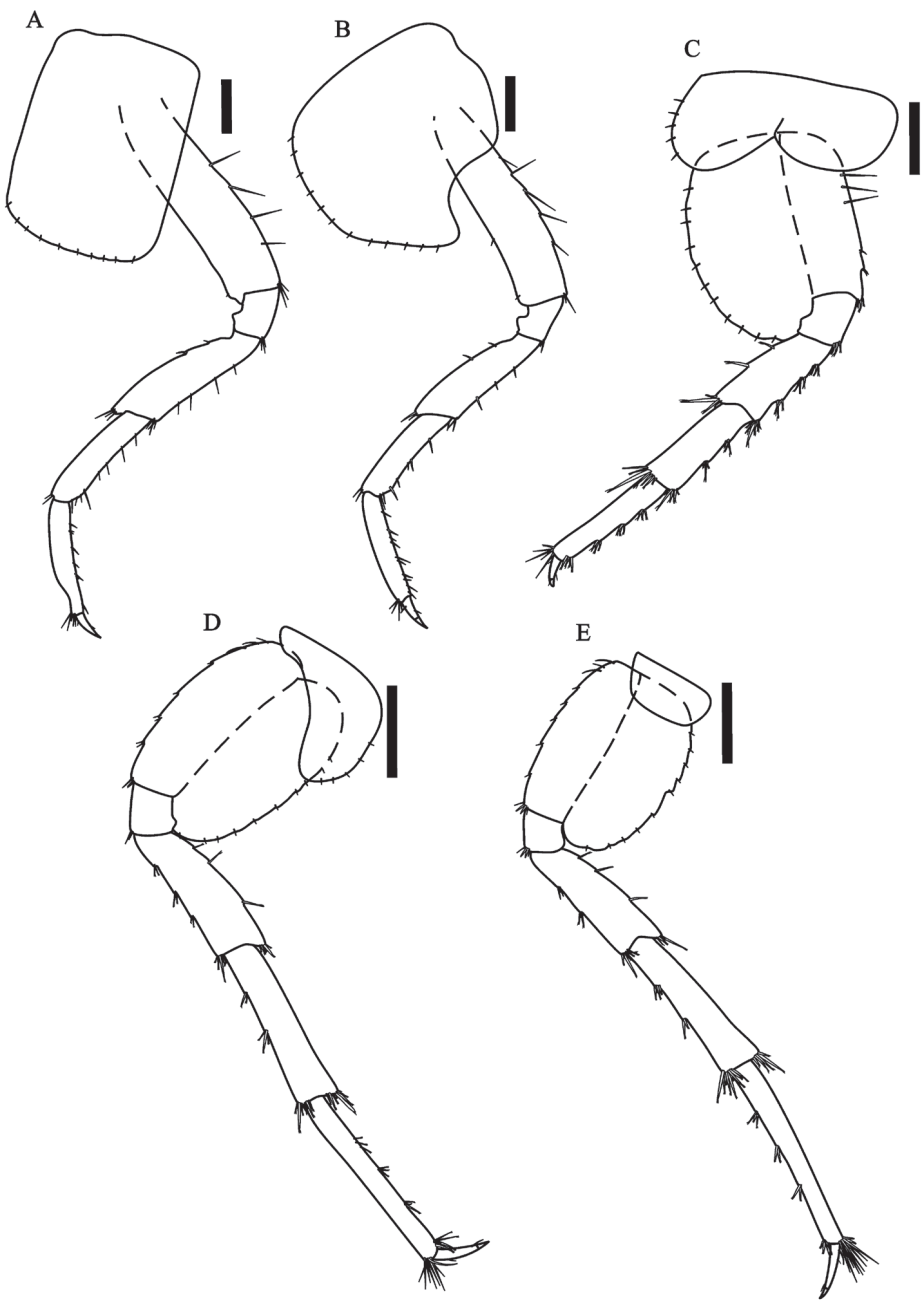
*Hyalella imbya* sp. n. Rodrigues and Bueno (male paratype, UFLA 0187). **A** peraeopod 3 **B** peraeopod 4 **C** peraeopod 5 **D** peraeopod 6 **E** peraeopod 7. Scale bars equals 200 µm for **A–E**.

**Figure 4. F4:**
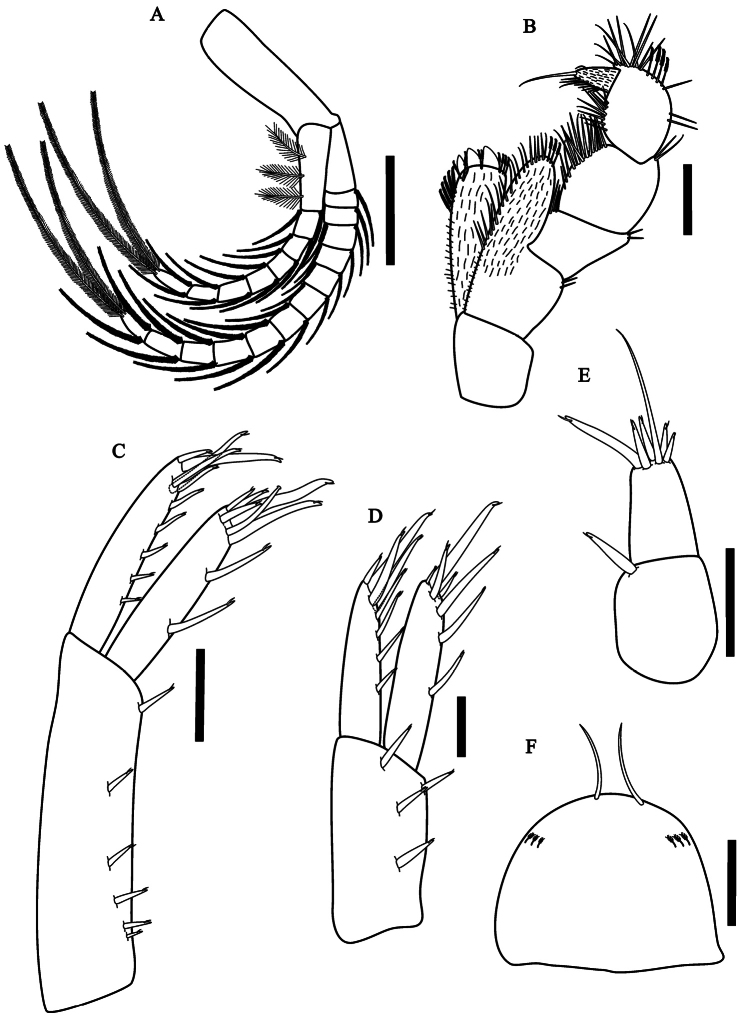
*Hyalella imbya* sp. n. Rodrigues and Bueno (male paratype, UFLA 0187). **A** pleopods **B** maxillipod **C** uropod 1 **D** uropod 2 **E** uropod 3 **F** telson. Scale bars equals 200 µm for **A**; 100 µm for **B–F**.

Peraeopods 3 (Fig. 3A) and 4 (Fig. 3B) merus and carpus posterior margin with clusters of simple setae; propodus posterior margin with six to seven groups of simple setae; dactylus less than half-length of propodus. Peraeopods 5 to 7 dactylus less than half-length of propodus; merus, carpus and propodus posterior margin with 4-5 marginal clusters of 2-9 cuspidate setae with an accessory setae. Peraeopod 3 sub-equal to peraeopod 4; peraeopod 5 (Fig. 3C) smaller than others; peraeopods 6 (Fig. 3D) and 7 (Fig. 3E) much longer than others. Coxal plates - peraeopod 3: longer than wide, width about half its length; peraeopod 4: wider than long; peraeopod 5: wider than long, with two lobes; peraeopod 6: ovate; peraeopod 7: wider than long. All coxal plates with simple setae on the border.

Pleopods (Fig. 4A) peduncle smaller than flagellum, without coupling spines; rami with several plumose setae; plumose setae of the last article longer 1.4 times than peduncle.

Uropod 1 (Fig. 4C) peduncle 1.7 times longer than rami; outer ramus longer than inner ramus; outer ramus with six cuspidate setae with an accessory seta, four cuspidate setae with an accessory seta apically, one smaller and three more longer, one of them with almost half length of the outer ramus; inner ramus with two dorsal cuspidate setae with an accessory seta on inner margin, male with a short curved seta apically on the ramus, five cuspidate setae with an accessory seta apically, three smaller and two more longer, one of them more than half of the length of the inner ramus; peduncle setation present.

Uropod 2 (Fig. 4D) shorter than uropod 1; ramus and peduncle of the same length; inner ramus with three dorsal setae and four distal setae, one more than half the length of the inner ramus; outer ramus with four dorsal setae and four distal setae, one more than half the length of the outer ramus; peduncle wider than ramus with four cuspidate setae with an accessory setae.

Uropod 3 (Fig. 4E) shorter than telson, shorter than peduncle of uropod 1 and uropod 2; inner ramus absent; outer ramus uniarticulate; peduncle longer than wide with one cuspidate seta with an accessory seta distally; ramus shorter than peduncle; basal width 1.8 times the width of ramus apex, with five cuspidate setae with an accesory seta and one long simple seta, longer than peduncle.

Telson (Fig. 4F) entire, apically rounded, more than 1.2 times wider than long, with two long simple apical setae; sometimes with plumose setae laterally.

Coxal gills sac-like present on pereonites 2 to 6. Sternal gills tubular present on pereonites 3 to 7.

##### Female.

Mean body length: 4.8 ± 0.43 mm, mean head length: 0.48 ± 0.03 mm (n=10) (Fig. 6C). Antenna 1 flagellum with 19 to 20 articles; antenna 2 similar in shape to male; flagellum with 15 to 16 articles.

**Figure 5.**
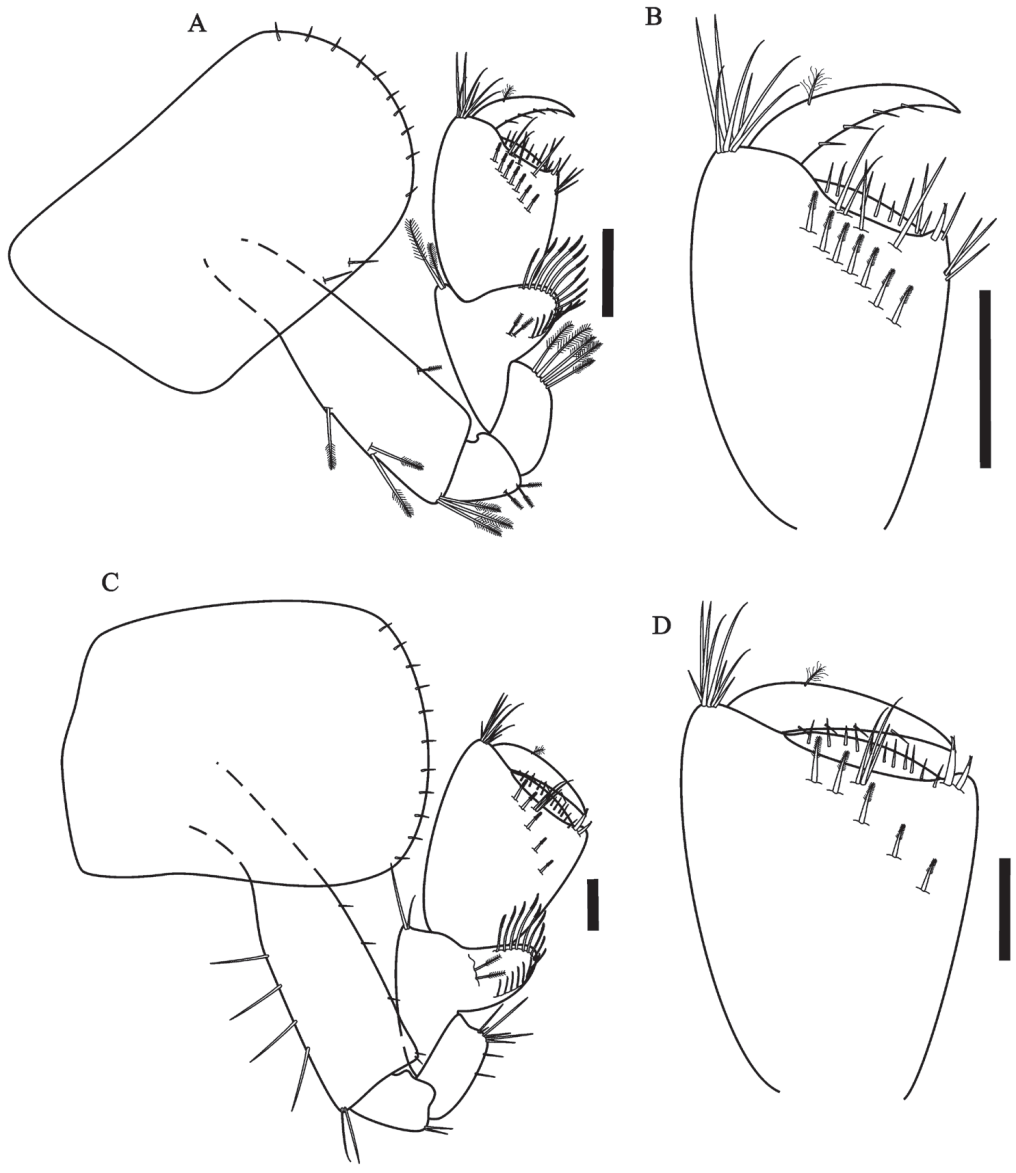
*Hyalella imbya* sp. n. Rodrigues and Bueno (female allotype, UFLA 0187). **A** gnathopod 1 **B **gnathopod 1 propodus and dactylus **C** gnathopod 2 **D** gnathopod 2 propodus and dactylus. Scale bars equals 100 µm for **A–D**.

Gnathopod 1 (Fig. 5A) similar in size and shape to gnathopod 2; without comb-scales; propodus (Fig. 5B) longer than wide; similar to male gnathopod 1 except that propodus is less narrow and shorter. Gnathopod 2 (Fig. 5C) different from male gnathopod 2 in shape and smaller; propodus (Fig. 5D) length 1.1 times maximum width, subchelate, inner face with five serrate setae, palm transverse, without comb-scales.

##### Etymology.

The specific name, *imbya*, honors the indigenous tribe Mbyá-Guarani that inhabited the local before the colonization of european immigrants.

##### Habitat.

Freshwater, hypothelminorheic.

##### Remarks.

The area where specimens of *Hyalella imbya* were collected was severely altered in the last recent years (Fig. 7). The area suffered during decades with agriculture, but the recent impacts were even more harmful. Such area is drained to a tributary stream which flows to Ijuí river (its margin was about 3.5 km far from the sampled area). In 2011 the riparian vegetation of this tributary was removed (Fig. 7B) and a reservoir was filled, flooding the deforested area (Fig. 7C). The phreatic level was altered since the distance between the sampled area and the nearest reservoir´s margin was reduced to about 2 km. In a visit maid on March 30, 2012 by two of the authors (S. G. Rodrigues and A. A. P. Bueno) to the same area no specimen was found. The area was completely dry and no spring was observed. It seems that changes in the hydrological parameters due to the building of São José reservoir altered the species habitat. Further considerations regarding such impacts will be discussed later.

**Figure 6.**
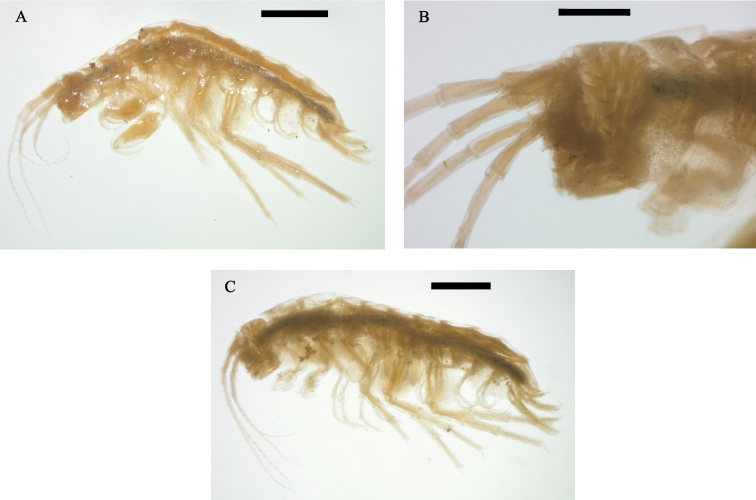
*Hyalella imbya* sp. n. Rodrigues and Bueno (male holotype, UFLA 0187). **A** male specimen fixed (body length = 4.8 mm, head length = 0.47 mm) **B** detail of the head showing the absence of eyes in the male specimen fixed **C** female specimen fixed (body length = 4.5 mm, head length = 0.45 mm). Scale bar equal 1 mm for **A**; 0.5 mm for **B**; 1 mm for **C**.

**Figure 7.**
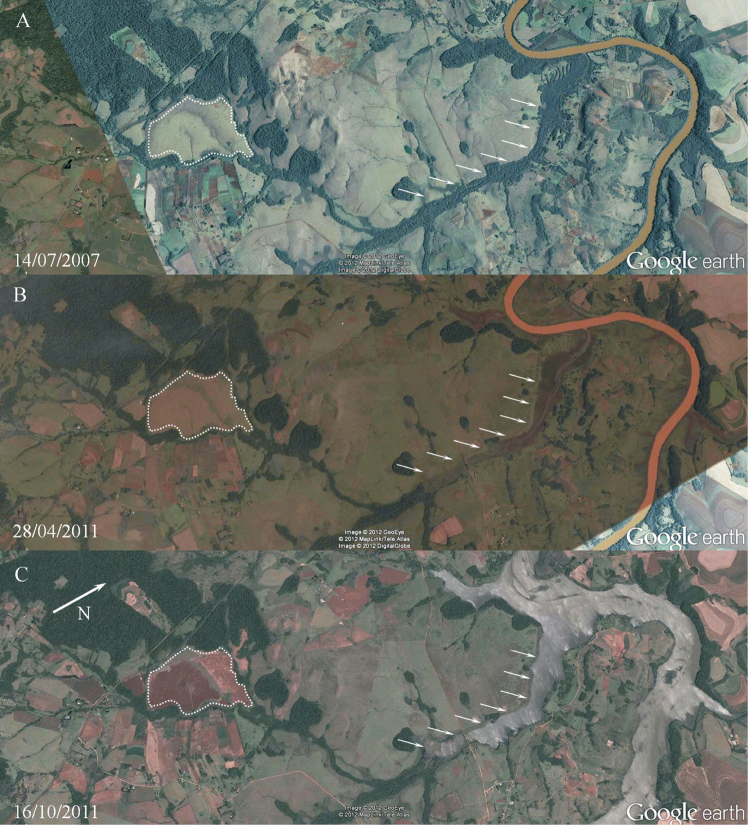
Habitat of *Hyalella imbya* sp. n. Rodrigues and Bueno. **A** The area bounded by a dotted line represents the region where specimens were found **B** the riparian vegetation was removed from a tributary stream (white arrows) from Ijuí river **C** the São José reservoir already filled.

## Discussion

### Affinities

There are four troglobiotic species of *Hyalella* described: *Hyalella anophthalma* Ruffo, 1957; *Hyalella caeca* Pereira, 1989, *Hyalella muerta* Baldinger, Shepard and Threloff, 2000, and *Hyalella spelaea* Bueno and Cardoso, 2011. Two of these, *Hyalella caeca* and *Hyalella spelaea*, occur in Brazil, both in caves of São Paulo, Southeastern Brazil (Cardoso et al. 2011). *Hyalella anophtalma* occurs in a cave at Venezuela (Ruffo 1957) and *Hyalella muerta* also occurs in subterranean environment in Death Valley National Park, California, USA (Baldinger et al. 2000).

*Hyalella imbya* has the first pair of antennae elongated, which is longer than the second pair, a characteristic previously observed only for *Hyalella muerta* (Table 1). However, the largest size of the antenna is more pronounced for *Hyalella imbya*, which presents many more articles than other troglobiotic species. The two species have sternal gills from pereonite 3 to 7, while others present such gills on pereonites 2 to 7.

**Table 1.** Characters of troglobiotic species of *Hyalella* (A1: Antenna 1; A2: Antenna 2; G1: Gnathopod 1; G2: Gnathopod 2; U1: Uropod 1).

Characters	*Hyalella anophthalma* Ruffo, 1957	*Hyalella caeca* Pereira, 1989	*Hyalella muerta* Baldinger, Shepard & Threloff, 2000	*Hyalella spelaea* Bueno & Cardoso, 2011	*Hyalella imbya* Rodrigues & Bueno, 2012
A1: No. of articles of flagellum	6	10	9	9	18-23
A2: No. of articles of flagellum	9	14	8	16	14-16
Proportion of A1 and A2	A1<A2	A1<A2	A1>A2	A1<A2	A1>A2
Body length (mm)	3.2	6.0	3.3	4.35	5.03
G1: Comb-scales in propodus	Present	Absent	Absent	Present	Absent
G1: No. of setae in the inner face	--	8	5	7	7
G2: Lobiform process of propodus on the palmar corner	Present	Present	Absent	Absent	Absent
U1: Curved seta in the inner ramus	Absent	Absent	Absent	Absent	Present
Sternal gills tubular	2–7	2–7	3–7	2–7	3–7
Telson	Absence of setae	Two short simple apical setae	Four long simple apical setae	Two short simple apical setae	Two long simple apical setae

*Hyalella imbya* does not present comb-scales on propodus of gnathopod 1 and 2, even as *Hyalella caeca* and *Hyalella muerta*. Similarly to *Hyalella muerta* and *Hyalella spelaea*, the new species does not have a lobiform process on the propodus of gnathopod 2. When compared to *H anophthalma*, it is possible to note that the two species do not exhibit characteristic in common, only the absence of eyes.

Moreover, *Hyalella imbya* also has particular characteristics that make it differ from other troglobiotic species: the presence of curved seta in the inner ramus of uropod 1; reduction in size of uropod 3 and posterior lobe in carpus of gnathopod 2; elongation of appendices, as the first pair of antennas and the pereopods 6 and 7.

### Conservation status

Problems concerning the hypothelminorheic habitat conservation were already discussed (Boulton 2005, 2009, Fiser et al. 2010). According to Fiser et al. (2010), the main threats to this habitat are land drainage and agricultural improvement. The superficial nature of the hypothelminorheic habitat allied to the restricted distribution of the species makes them especially vulnerable to impacts (Culver et al. 2006). In South America, such habitats have been completely neglected and consequently not studied.

The region where the new species was found represents a very threatened type of ecosystem, the wetlands. This ecosystem presents a high diversity, with high levels of endemism, and is among the most productive environments of the world. Such traits make the wetlands priority ecosystem for conservation (Barbier et al. 1997, Mitsch and Gosselink 2000).

About half of South America wetlands are located in Brazil, and the state of Rio Grande do Sul has the greatest record for these ecosystems: 3.441 areas covering 10.7% of the total area of the state (Maltchik et al. 2003). Despite their great environmental importance, these ecosystems are among the most threatened habitats in the world (Mitsch and Gosselink 2000).

It is estimated that 90% of these areas in Rio Grande do Sul have already disappeared due to urban development, construction of dams and reservoirs, as well as expansion of areas of agriculture, especially rice and soybean, causing fragmentation and deterioration of these ecosystems (Maltchik and Rolon 2010).

The agricultural expansion is one of the main factors that affect and hinder the conservation of wetlands. The soil of these ecosystems can produce up to 50 times more vegetal organic matter area then a similar natural field, and eight times more than a cultivated field, which makes them targets for growing crops of economic importance, such as rice (Barbier et al. 1997). The state of Rio Grande do Sul is the leading producer of rice in Brazil. This type of plant has a dynamic water regime, with variations between terrestrial and aquatic stages. Since the remaining surface of the culture can stay up to two years without water, this can cause a great impact to the community of plants and animals, as well as throughout the landscape as a whole (Maltchik and Rolon 2010).

The severe impacts caused by the bulding of São José reservoir apparently altered the hypothelminorheic habitat of the area, furthermore nowadays the site of occurrence of *Hyalella imbya* is inserted in a large area of soybean farming. Since only a single visit was made to the area after such impacts, it is impossible to assess the real status of the species and its habitat. Hypothelminorheic habitats are characterized by persistent wet spots, but this condition is no longer observed in the area. The species may eventually be associated from subterranean habitats at the present moment, but one cannot discard the possibility of its extinction. We can assume minimally that it is critically threatened at the moment.

The Brazilian laws concerning cave fauna has been recently altered. Although it is somewhat restrictive in some cases (assuring the protection of rare troglobiotic species), it obviously lacks a broader conception of subterranean habitats. Accordingly, it is important to incorporate in such laws the protection of shallow subterranean habitats, especially those strongly threatened, as the hypothelminorheic.

## Supplementary Material

XML Treatment for Hyalella imbya
